# Prostate-specific antigen velocity as a predictor of survival outcomes in patients with prostate cancer: a meta-analysis

**DOI:** 10.3389/fonc.2026.1656688

**Published:** 2026-02-10

**Authors:** Feilun Cui, Yueshi Zhang, Ziyan Liu, Yongjing Zhou, Changfeng Man, Yu Fan

**Affiliations:** 1 Department of Urology, The Fourth Affiliated Hospital of Jiangsu University, Zhenjiang, China; 2 Department of Urology, Affiliated Taizhou Second People’s Hospital of Yangzhou University, Taizhou, China; 3 Department of Medical Laboratory Science, Liaoning University of Traditional Chinese Medicine, Shenyang, Liaoning, China; 4 Cancer Institute, The Affiliated People’s Hospital, Jiangsu University, Zhenjiang, China

**Keywords:** all-cause mortality, meta-analysis, prostate cancer, prostate cancer–specific mortality, prostate-specific antigen velocity

## Abstract

**Background:**

Prostate-specific antigen velocity (PSAV) has emerged as a promising biomarker for predicting survival outcomes in prostate cancer patients.

**Objectives:**

To assess the association between PSAV and both all-cause mortality and prostate cancer-specific mortality (PCSM) in men diagnosed with prostate cancer by conducting a meta-analysis.

**Methods:**

A comprehensive search of electronic medical databases, including PubMed, Web of Science, and Embase, was conducted to identify relevant studies published up to March 1, 2025. Studies reporting adjusted hazard ratios (HR) with 95% confidence intervals (CI) for survival outcomes in prostate cancer patients, based on categorical analyses of PSAV, were included. Pooled HRs with 95% CI was calculated using random-effects models to account for clinical heterogeneity across studies.

**Result:**

Eleven studies involving 3,713 prostate cancer patients were identified and analyzed. The meta-analysis revealed that elevated PSAV was associated with a higher risk of both all-cause mortality (HR 1.96; 95% CI 1.33–2.88) and PCSM (HR 5.38; 95% CI 2.76–10.51). In stratified analyses, the pooled HR for all-cause mortality was 1.91 (95% CI 1.43–2.55) among patients with localized prostate cancer, compared to 1.26 (95% CI 0.41–3.84) in those with metastatic disease.

**Conclusions:**

Elevated PSAV is a significant predictor of all-cause mortality and PCSM in prostate cancer patients. Measuring PSAV has the potential to improve the prediction of survival outcomes in this population. However, further research is needed to standardize PSAV measurement and validate its predictive value across diverse patient groups. Systematic review registration.

## Introduction

Prostate cancer is one of the most frequently diagnosed malignancies in 2022, accounting for 7.3% of all cancers globally (1). This condition continues to pose an increasing burden in both China and the United States (2). The 5-year survival rate for metastatic prostate cancer is approximately 30% (3). Traditionally, the combination of prostate-specific antigen (PSA) levels, Gleason score, and cancer stage has been utilized for the risk classification of prostate cancer (4). However, accurately predicting disease progression and survival outcomes remains a significant clinical challenge, underscoring the urgent need to enhance the risk stratification of prostate cancer.

PSA has long been a cornerstone in the diagnosis and management of prostate cancer. However, its utility is limited by variability in individual patient contexts and the influence of non-cancerous conditions, such as benign prostatic hyperplasia (5). PSA kinetics refers to the changes in PSA levels over time and is used as a dynamic marker to assess prostate cancer risk, treatment response, and disease progression. Prostate-specific antigen velocity (PSAV), PSA doubling time (time required for PSA levels to double), and PSA decline rate (rate of PSA reduction) are the three core kinetic parameters. PSAV is the rate of change in serum PSA level over time, expressed in nanograms per milliliter per year (6). Several studies have suggested that rapid increases in PSA levels were associated with biochemical recurrence or progression (7–9), metastasis (10, 11), and poorer survival outcomes (8, 9, 12). However, the results have been inconsistent (13, 14), partly due to variations in study design, patient populations, and PSAV thresholds.

A comprehensive synthesis of the available evidence is therefore necessary to clarify the prognostic value of PSAV in patients diagnosed with prostate cancer. This meta-analysis aims to thoroughly evaluate the association between PSAV and both all-cause mortality and prostate cancer-specific mortality (PCSM) within this patient population.

## Materials and methods

### Literature search

The reporting of this study adhered to the Meta-analysis of Observational Studies in Epidemiology (15) and the Preferred Reporting Items for Systematic Reviews and Meta-analyses guidelines (16). A comprehensive search was conducted across multiple electronic databases, including PubMed, Embase, and Web of Science, up to March 1, 2025, to identify relevant studies. The search strategy utilized a combination of the following keywords without language restrictions: (“prostate cancer” OR “prostate carcinoma”) AND “prostate-specific antigen velocity “ AND (“death” OR “mortality” OR “survival”). The detailed search strategy is provided in **Supplementary Text S1**. Additionally, the reference lists of retrieved studies and relevant reviews were manually screened to identify any potentially eligible studies for inclusion.

### Study selection

Eligible studies met the following criteria:1) population: patients diagnosed with prostate cancer; 2) Exposure: PSAV reported through categorical analysis; 3) Comparison: patients with higher versus lower PSAV levels; 4)Outcome measures: all-cause mortality and PCSM; 5) Study design: retrospective or prospective cohort studies; and 6) Reporting hazard ratios (HR) for the association between PSA velocity and survival outcomes, adjusted for at least age or baseline PSA, along with corresponding 95% confidence intervals (CI). For multiple studies with overlapping participant populations, only the article with the most comprehensive data was included. Exclusion criteria were as follows: 1) Reporting relative risk based on continuous analysis of PSAV; 2) Providing unadjusted relative risk; 3) Lacking relative risk data; and 4) Reviews or conference abstract. Two reviewers independently conducted the literature search, screened titles and abstracts, and retrieved full-text articles to assess eligibility. Any disagreements were resolved through consensus or by consulting the corresponding author.

### Data extraction and quality assessment

Two independent reviewers extracted data from the eligible studies using a pre-designed data extraction form. The extracted data included the first author’s name, publication year, geographical region, study design, prostate cancer subtypes, sample size, age distribution, treatment modalities, threshold for elevated PSAV, follow-up duration, multivariable-adjusted HR with corresponding 95% CIs for survival outcomes, and the covariates adjusted for in the multivariable analysis. The quality of the included studies was assessed using the Newcastle - Ottawa Scale (NOS) for cohort studies (17). Studies with an NOS score of 7 or higher were classified as high-quality, while those with scores between 4 and 6 were considered moderate quality. Any discrepancies in data extraction or quality assessment were resolved through consensus or by consulting the corresponding author.

### Data analysis

The combined adjusted hazard ratio (HR) and 95% confidence interval (CI) were used to evaluate the association between elevated PSAV and both all-cause mortality and prostate cancer-specific mortality (PCSM). Heterogeneity across studies was assessed using the I² statistic, with an I² value greater than 50% indicating substantial heterogeneity. Due to evident clinical heterogeneity among the studies, a random-effects model was selected for all pooled analyses, regardless of heterogeneity levels. Pre-specified subgroup analyses were conducted based on patient age (≥ 65 years or <65 years), sample size (≥ 300 or <300), cancer subtype (metastatic prostate cancer or localized prostate cancer), and follow-up duration (≥ 5 years or <5 years). Sensitivity analyses were performed to assess the robustness of the results by sequentially excluding individual studies. Publication bias was evaluated using Begg’s test (18) and Egger’s test (19). All statistical analyses were performed using Stata 12.0 software.

## Results

### Search results

**Figure 1:**
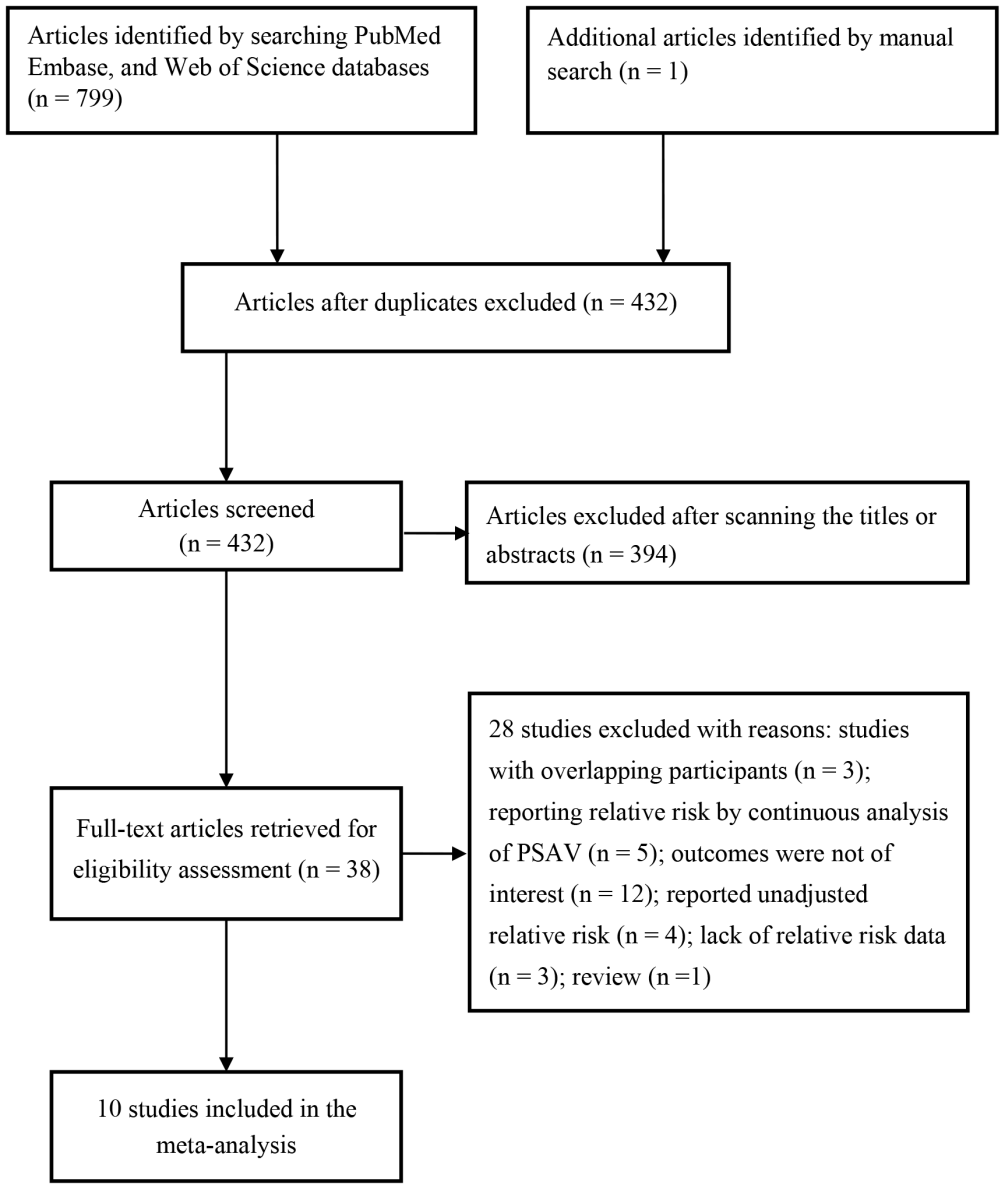
Flow chart of studies selection process.

A comprehensive search of electronic databases, supplemented by a manual search, initially identified 799 publications from the databases and 1 additional article. After removing duplicates, 432 articles remained for further screening. Following a review of titles and abstracts, 394 articles were excluded. The remaining 38 articles were retrieved for a full-text eligibility assessment. After applying predefined inclusion and exclusion criteria, 28 articles were excluded, leaving 10 studies (8, 9, 12–14, 20–24) for final analysis (**Figure 1**).

### Study characteristics

The key characteristics of the included studies are summarized in **Table 1**. These studies, published between 2004 and 2022, were all retrospective in design and conducted across multiple countries, including the United State (8, 9, 12, 20, 24), Canada (13), Spain (22), Australia (23), and China (14, 21). The sample sizes ranged from 91 to 1,095, encompassing a total of 3,713 patients. The mean/median age of the patients ranged from 60 to 71.2 years old. PSAV cutoff values were reported in either ng/mL/month or ng/mL/year, with significant heterogeneity in the cutoff values across studies. The follow-up ranged between 1.25 and 6.9 years. According to the NOS criteria, two studies (12) were deemed moderate quality, while the remaining studies were rated as high-quality (**Supplementary Table S1**).

**Table 1:** Characteristics of the included studies.

Author/year	Design	Region	Patients	Age (years)	Treatment modalities	Cutoff value of PSAV	Follow-up (years)	Outcomes HR (95% CI)	Covariates adjusted
D’Amico 2004 (8)	R	USA	Localized PC 1,095	Median 65.4	RP	PSAV >2 ng/mL/year	5.1	PCSM 9.8 (2.8-34.3) ACM 1.9 (1.2-3.2)	Age at diagnosis, tumor stage at diagnosis, Gleason score, PSA level
D’Amico 2005 (9)	R	USA	Localized PC 358	Median 71.2	Radiotherapy	PSAV >2 ng/mL/year	4.0	PCSM 12.0(3.0-54.0) ACM 2.1(1.3-3.6)	Age at diagnosis, clinical tumor category, Gleason score, PSA level
Rozhansky 2006 (12)	R	USA	HRMPC 213	NP	Chemotherapy	PSAV > 0.0 ng/mL/month	1.25	ACM 1.84 (1.31-2.58)	LDH, Hb, ECOG PS, PSA
Daskivich 2007 (20)	R	USA	PC 91	Median 60	Chemotherapy	PSAV >10 ng/mL/year	2.8	ACM 2.8 (1.5-5.3)	PSADT, PSA nadir during ADT of >0.2 ng/mL, Hb, treatment
Palma 2008 (13)	R	Canada	Intermediate- high risk PC 277	Median 70	ADT, Radiotherapy	PSAV > 3 ng/mL/month	6.8	PCSM 2.75(1.27-5.95) ACM 1.70(0.99-2.92)	Age, Gleason score
Ma 2009 (21)	R	China	mPC 250	Median 70	ADT	PSAV > 9.1 ng/mL/year	2.0	ACM 2.92 (1.13-7.55)	Age, PSA, Gleason score, M1c stage, PSADT
Rodríguez-Alonso 2010 (22)	R	Spain	PC 265	64.6 ± 5.5	RP	PSAV >3 ng/mL/year	4.3	ACM 22.8 (4.71-90.5)	Age, PSA, combined Gleason of specimen, surgical margins, extracapsular disease
Shi 2013 (23)	R	Australia	Localized PC 848	69.6 ± 7.3	ADT, Radiotherapy	PSAV >0.2 ng/mL/year	5.5	PCSM 5.15(1.99-13.30) ACM 1.74 (1.04-2.90)	Age, Gleason score, PSA at diagnosis, adjuvant/neoadjuvant ADT use within 2 years
Suzman 2015 (24)	R	USA	BRPC 196	Median 63	Non-hormonal agents	PSAV > 0.59 ng/mL/month	6.9	ACM 2.33 (1.28-4.35)	Age, Gleason score, pre-metastatic use of ADT
Wang 2022 (14)	R	China	mCRPC 120	NP	Abiraterone	PSAV >0.77 ng/mL/month	NP	ACM 0.41 (0.23-0.73)	Baseline PSA at abiraterone use, lowest PSA, painkiller, ALP

HR, hazard ratio; CI, confidence interval; NP, not provided; R, retrospective; mCRPC, metastatic castration-resistant prostate cancer; HRMPC, hormone-refractory metastatic prostate carcinoma; BRPC, biochemically-recurrent prostate cancer; PC, prostate cancer; PSA, prostate-specific antigen; PSAV, prostate-specific antigen velocity; PSADT, prostate-specific antigen doubling time; ECOG, Eastern Cooperative Oncology Group; PS, performance status; BCF, biochemical failure ADT, androgen-deprivation therapy; Hb, hemoglobin; AKP, alkaline phosphatase; ALP, alkaline phosphatase; LDH, lactate dehydrogenase; BMI, body mass index; OS, overall survival; RP, radical prostatectomy.

### All-cause mortality

**Figure 2:**
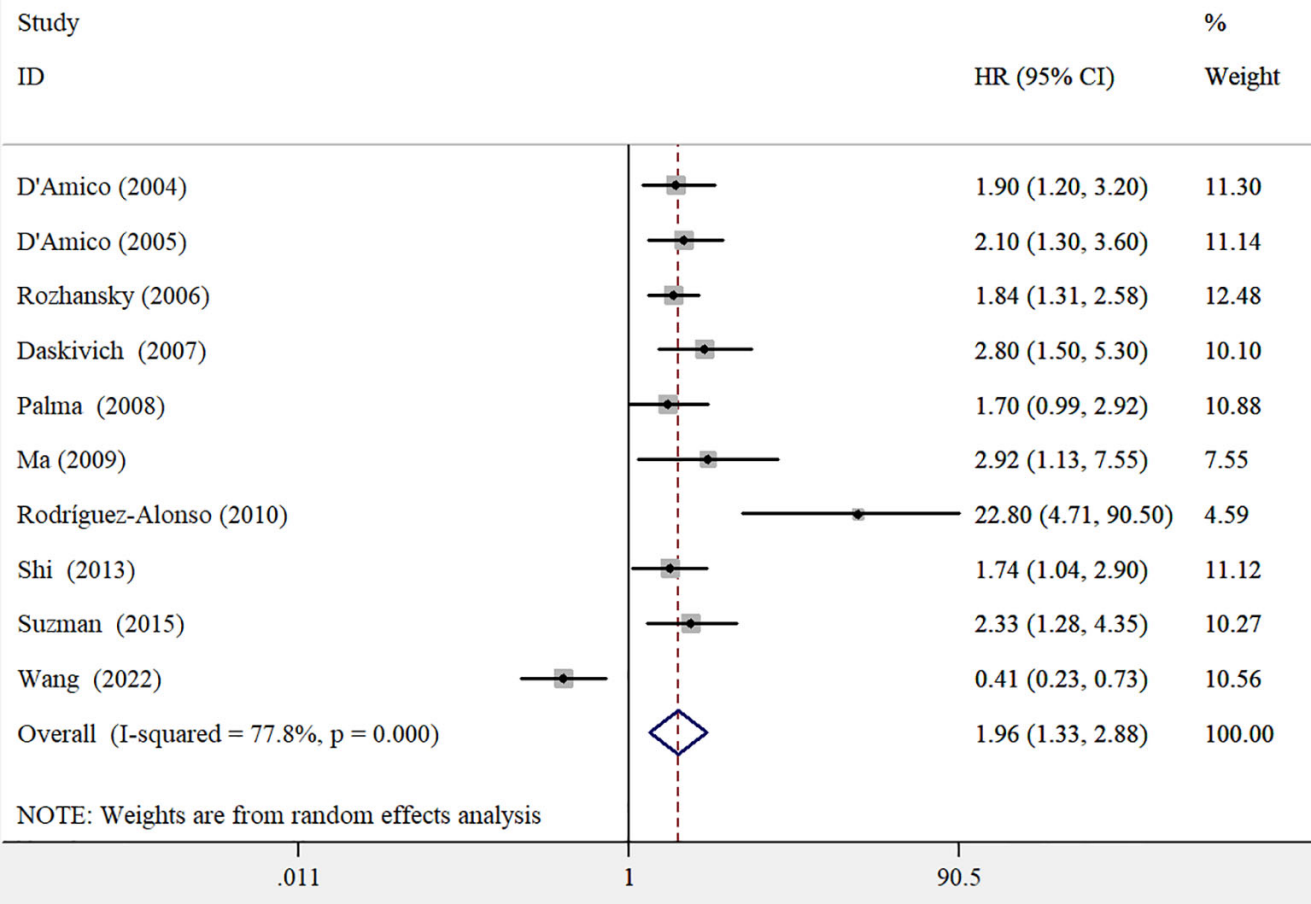
Pooled adjusted hazard ratios with 95% confidence intervals of all-cause mortality for higher versus reference lower prostate-specific antigen velocity.

All included studies examined the association between PSAV and all-cause mortality. The overall findings demonstrated that elevated PSAV was associated with a higher risk of all-cause mortality (HR 1.96; 95% CI 1.33–2.88; **Figure 2**), although significant heterogeneity was observed across studies (*I*^2^ = 77.8%, *p* < 0.001). No publication bias was detected, as indicated by Egger’s test (*p* = 0.278) and Begg’s test (*p* = 0.107). A leave-one-out sensitivity analysis confirmed the credibility of the pooled risk estimate. In subgroup analyses (**Table 2**), the pooled HR for all-cause mortality was 1.91 (95% CI 1.43–2.55) among patients with localized prostate cancer, compared to 1.26 (95% CI 0.41–3.84) in those with metastatic disease. Additionally, the association between elevated PSAV and all-cause mortality was stronger in studies with follow-up durations of less than 5 years (HR 2.76; 95% CI 1.69–4.52) and in studies where the median/mean patient age was ≤65 years (HR 4.10; 95% CI 1.61–10.44).

**Table 2:** Subgroup analysis on all-cause mortality.

Subgroups	No. of studies	Pooled adjusted HR	95%CI	Heterogeneity between studies
Sample sizes				
≥ 300	3	1.91	1.43-2.55	*p* = 0.878; *I*^2^ = 0.0%
< 300	7	2.10	1.14-3.84	*p* < 0.001; *I*^2^ = 85.0%
Cancer subtypes				
Localized PC	3	1.91	1.43-2.55	*p* = 0.878; *I*^2^ = 0.0%
Metastatic PC	3	1.26	0.41-3.84	*p* < 0.001; *I*^2^ = 91.0%
Age at baseline				
≥ 65 years	5	1.92	1.50-2.46	*p* = 0.877; *I*^2^ = 0.0%
< 65 years	3	4.10	1.61-10.44	*p* = 0.019; *I*^2^ = 74.8%
Follow-up duration				
≥ 5 years	4	1.88	1.44-2.45	*p* = 0.874; *I*^2^ = 0.0%
< 5 years	5	2.76	1.69-4.52	*p* = 0.021; *I*^2^ = 65.5%

HR, hazard ratio; CI, confidence intervals; PC, prostate cancer.

### Prostate cancer-specific mortality

**Figure 3:**
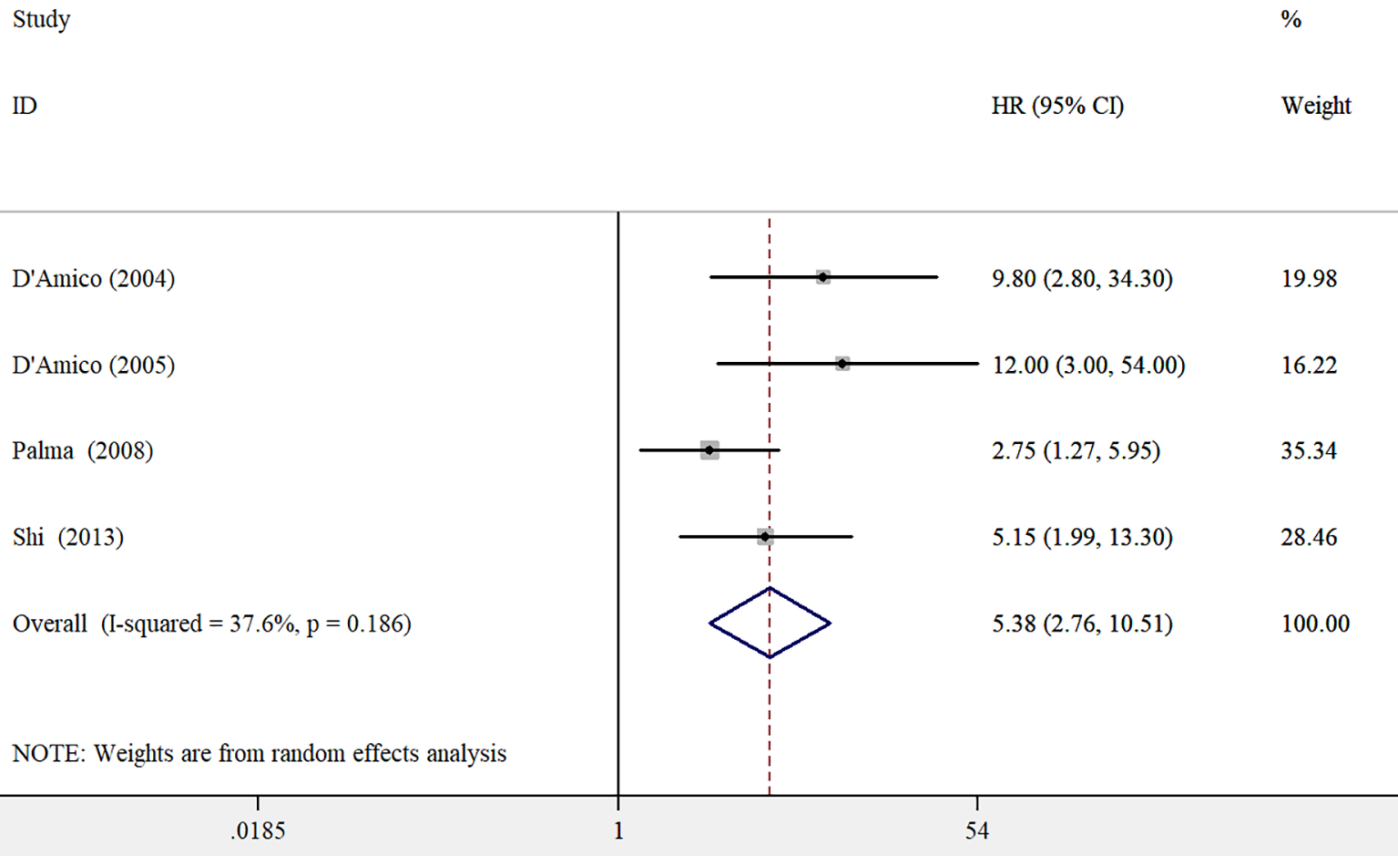
Pooled adjusted hazard ratios with 95% confidence intervals of prostate cancer-specific mortality for higher versus lower prostate-specific antigen velocity.

A total of 4 studies (8, 9, 13, 23) investigated the association between PSAV and PCSM. The overall findings revealed that elevated PSAV was associated with a significantly higher risk of PCSM (HR 5.38; 95% CI 2.76–10.51; **Figure 3**), despite notable heterogeneity across studies (*I*^2^ = 37.6%, *p* = 0.186). A leave-one-out sensitivity analysis confirmed the robustness of the pooled risk estimate.

## Discussion

This meta-analysis evaluated the association between PSAV and survival outcomes in patients with prostate cancer, specifically focusing on all-cause mortality and PCSM. The findings indicate that an increased PSAV is a significant predictor of both all-cause mortality and PCSM. Compared to patients with lower PSAV, those with higher PSAV exhibited a 96% increased risk of all-cause mortality and a 5.38-fold increased risk of PCSM. These results underscore the potential of PSAV as a valuable biomarker for risk stratification and survival prediction in patients with prostate cancer.

Subgroup analyses provided further insights into the relationship between PSAV and survival outcomes. Among patients with localized prostate cancer, the association between increased PSAV and all-cause mortality was robust; however, this association was not statistically significant in patients with metastatic disease. This discrepancy may be attributed to the advanced nature of metastatic prostate cancer, where factors such as tumor burden, treatment resistance, and comorbidities may overshadow the predictive value of PSAV. Additionally, the stronger association between PSAV and all-cause mortality in studies with shorter follow-up durations and younger patient populations suggests that PSAV may be particularly relevant in earlier stages of the disease or among patients with a longer life expectancy, where timely interventions could significantly influence outcomes. The results also demonstrated a significant association between elevated PSAV and PCSM, underscoring the potential of PSAV as a biomarker for aggressive disease and an increased risk of death specifically attributable to prostate cancer. However, the limited number of studies reporting PCSM outcomes emphasizes the need for further research to validate these findings.

The pooled estimate of all-cause mortality should be interpreted in the context of significant heterogeneity. Most notably, the extreme HR reported by Rodríguez-Alonso et al., 2010 (22) is a potential outlier. However, we conducted a sensitivity analysis by excluding this study and confirm that the overall direction and statistical significance of our pooled results remained unchanged (HR 1.73; 955 CI 1.23-2.44), which supports the robustness of our primary findings.

In addition to the categorical analysis of PSAV, elevated PSAV was also a significant predictor of worse survival outcomes when analyzed as a continuous variable. Among 201 men with nonmetastatic castration-resistant prostate cancer, each logarithmic increase in PSAV (ng/mL/year) was associated with a 39% reduction in overall survival (25). Furthermore, in a cohort of 915 patients with metastatic prostate cancer, each unit increase in PSAV was linked to a 5.23-fold higher risk of death (26).Additionally, higher PSAV was identified as a significant prognostic biomarker for overall survival in patients with metastatic castration-resistant prostate cancer (adjusted HR 1.008; 95% CI 1.004–1.012) (27) and hormone-refractory prostate cancer (adjusted HR 1.004; 95% CI 1.001–1.007) (28). These findings provide further evidence supporting the role of increased PSAV in predicting overall survival in prostate cancer patients.

The results of this meta-analysis have significant clinical implications. Determining PSAV could potentially enhance the prediction of overall survival in prostate cancer patients, enabling more personalized treatment strategies. The strong association between increased PSAV and PCSM further underscores the importance of PSAV as a prognostic biomarker. This strong correlation indicates that PSAV can effectively predict the risk of death specifically from prostate cancer, which is crucial for determining the intensity of treatment and the necessity for closer surveillance. However, several areas warrant future research: 1) establishing a standardized protocol for PSAV measurement, which specifies core requirements including a minimum of three measurements collected over 18–24 months, standardized sampling time points, PSAV calculation via linear regression analysis, and an explicitly defined measurement timeframe, would improve the comparability of future studies and enhance the reliability of PSAV as a biomarker; 2) validating its predictive value across diverse patient populations; 3) exploring the optimal PSAV threshold for identifying high-risk patients; and 4) evaluating the incremental value of PSAV when combined with other prognostic markers and risk stratification tools.

Our meta-analysis has several limitations that warrant consideration. First, all included studies were retrospective in nature, which introduces potential selection bias and recall bias. Second, there was significant heterogeneity across studies regarding patient populations, PSAV measurement techniques or cutoff values, treatment approaches, and follow-up durations, all of which could introduce bias. Third, the methods for calculating PSAV varied among studies. Different intervals between PSA measurements, as well as diverse statistical approaches, can lead to inconsistent results. This lack of standardization in measuring PSAV undermines its reliability as a universal prognostic biomarker. In particular, interpreting PSAV should be done in the context of non-cancerous conditions, as these factors can cause fluctuations in PSA levels, making it challenging to accurately assess the true velocity. Fourth, the results of subgroup analyses were based on a relatively small number of studies, and the interpretation of these findings should be approached with caution. Finally, different units and varying time frames (e.g., ng/mL/year, log[ng/mL]/year, or ng/mL/month) rendered quantitative pooling of continuous PSAV results impossible, which reinforce the need for standardized continuous PSAV reporting guidelines in future research.

## Conclusions

This meta-analysis provides robust evidence that elevated PSAV is a significant predictor of both all-cause mortality and PCSM in prostate cancer patients. These findings highlight the potential clinical utility of incorporating PSAV into routine practice to improve risk stratification and enhance the prediction of survival outcomes, particularly in patients with localized diseases. However, further research is needed to standardize PSAV measurement methods and validate its predictive value across diverse patient populations.

## Data Availability

The original contributions presented in the study are included in the article/**Supplementary Material**. Further inquiries can be directed to the corresponding authors.
