# Supplementary material for: Prostate-specific antigen velocity as a predictor of survival outcomes in patients with prostate cancer: a meta-analysis
Source: Front Oncol. 2026 Feb 10;16:1656688. doi: 10.3389/fonc.2026.1656688 (PMC12929101; doi:10.3389/fonc.2026.1656688)
Supplement: Supplementary file 1 [file DataSheet1.docx]

**Supplemental Text S1 Search strategy**

| 1. **PubMed** | **Query** | **Items found** |
| --- | --- | --- |
| #1 | Search: **Prostate-specific antigen velocity** | 761 |
| #2 | Search: **((prostate cancer) OR (prostate carcinoma)) OR (prostate neoplasm)** | 228,103 |
| #3 | Search:**((mortality) OR (death)) OR (survival)** | 3,660,265 |
| #4 | Search: **((#1) AND (#2)) AND (#3)** | **215** |

| **2. Embase** | **Query** | **Items found** |
| --- | --- | --- |
| #1 | Search'prostate-specific antigen velocity' OR ('prostate specific' AND ('antigen'/exp OR antigen) AND ('velocity'/exp OR velocity)) | 1,203 |
| #2 | Search'prostate cancer'/exp OR 'prostate cancer' OR (('prostate'/exp OR prostate) AND ('cancer'/exp OR cancer)) | 380,556 |
| #3 | Search 'prostate carcinoma'/exp OR 'prostate carcinoma' OR (('prostate'/exp OR prostate) AND ('carcinoma'/exp OR carcinoma)) | 96,807 |
| #4 | Search 'prostate neoplasm'/exp OR 'prostate neoplasm' OR (('prostate'/exp OR prostate) AND ('neoplasm'/exp OR neoplasm)) | 366,622 |
| #5 | Search #2 OR #3 OR #4 | 392,806 |
| #6 | Search 'mortality'/exp OR mortality | 2,159,261 |
| #7 | Search 'death'/exp OR death | 3,150,939 |
| #8 | Search'survival'/exp OR survival | 2,450,110 |
| #9 | Search #6 OR #7 OR #8 | 5,360,718 |
| #10 | Search #1 AND #5 AND #9 | 344 |
| #11 | Search #10 AND (**'**article**'**/it OR **'**article in press**'**/it) | **176** |

|  |  |  |
| --- | --- | --- |
| **3.** **Web of Science** | **Query** | **Items found** |
| **#1** | Search**Prostate-specific antigen velocity**(Topic) | 1,173 |
| **#2** | Search**prostate cancer**(Topic) or**prostate carcinoma**(Topic) or **prostate neoplasm**(Topic) | 377,697 |
| **#3** | **Searchmortality**(Topic) or**death**(Topic) or**survival**(Topic) | 5,141,334 |
| **#5** | **Search #1 AND #2 AND #3 AND #4 and Article**(Document Types) | **409** |
