# Supplementary material for: Prostate-specific antigen velocity as a predictor of survival outcomes in patients with prostate cancer: a meta-analysis
Source: Front Oncol. 2026 Feb 10;16:1656688. doi: 10.3389/fonc.2026.1656688 (PMC12929101; doi:10.3389/fonc.2026.1656688)
Supplement: Supplementary file 2 [file Table1.doc]

~~Supplementary Table S1 Quality assessment using the Newcastle–Ottawa Scale~~

| First author/Year | Representativeness of the exposed cohort | Selection of the non-exposed cohort | Ascertainment of exposure | Demonstration that outcome was not present at study start | Comparability of cohorts based on the design or analysis | Assessment of outcome | Enough follow-up periods# | Adequacy of follow-up of cohorts | Total scores |
| --- | --- | --- | --- | --- | --- | --- | --- | --- | --- |
| D’Amico 2004 (8) | ★ | ★ | ★ | ★ | ★★ | ★ | ★ | ★ | 9 |
| D’Amico 2005 (9) | ★ | ★ | ★ | ★ | ★★ | ★ |  | ★ | 9 |
| Rozhansky 2006 (12) |  | ★ | ★ | ★ | ★ | ★ |  | ★ | 6 |
| Daskivich 2007 (20) | ★ | ★ | ★ | ★ | ★★ | ★ |  | ★ | 8 |
| Palma 2008 (13) |  | ★ | ★ | ★ | ★ | ★ | ★ | ★ | 7 |
| Ma 2009 (21) | ★ | ★ | ★ | ★ | ★ | ★ | ★ |  | 7 |
| Rodríguez-Alonso 2010 (22) | ★ | ★ | ★ | ★ | ★★ | ★ |  | ★ | 8 |
| Shi 2013 (23) | ★ | ★ | ★ | ★ | ★★ | ★ | ★ | ★ | 9 |
| Suzman 2015 (24) |  | ★ | ★ | ★ | ★ | ★ | ★ | ★ | 7 |
| Wang 2022 (14) | ★ | ★ | ★ | ★ | ★ | ★ |  |  | 6 |

^#^ ≥2 years for metastatic prostate cancer and ≥ 5 years for others
